# Vitamin K Deficiency Presenting in an Infant with an Anterior Mediastinal Mass: A Case Report and Review of the Literature

**DOI:** 10.1155/2017/7628946

**Published:** 2017-02-09

**Authors:** Mauricio A. Palau, Amanda Winters, Xiayuan Liang, Rachelle Nuss, Susan Niermeyer, Megan Gossling, Clyde Wright

**Affiliations:** School of Medicine, University of Colorado, Boulder, CO, USA

## Abstract

We report a case of a 1-month-old infant with spontaneous thymic hemorrhage secondary to severe vitamin K deficiency. He was brought to medical attention due to scrotal bruising and during evaluation was noted to be tachypneic and hypoxemic. Chest X-ray revealed an enlarged cardiothymic silhouette, and a follow-up echocardiogram revealed a mass in the anterior mediastinum. Routine laboratory work-up revealed severe coagulopathy. Further questioning revealed the patient had not received prophylactic vitamin K at birth. The coagulopathy resolved with administration of vitamin K, and a biopsy confirmed the anterior mediastinal mass was due to spontaneous thymic hemorrhage.

## 1. Introduction

Vitamin K deficiency bleeding (VKDB), formerly known as hemorrhagic disease of the newborn, is a coagulopathy infants acquire due to insufficient vitamin K stores. Vitamin K is necessary for the synthesis of functional forms of coagulation factors II, VII, IX, and X in the liver [1]. These proteins are secreted into the blood and ultimately assist in the conversion of fibrinogen to fibrin and the formation of a hemostatic thrombus. Therefore, infants who accumulate inactive vitamin K-dependent coagulation factors have an increased bleeding tendency [2]. Herein, we report the case of a 1-month-old infant whose VKDB presented as scrotal bruising and respiratory distress due to an anterior mediastinal mass that was ultimately determined to be spontaneous thymic hemorrhage, representing an unusual clinical manifestation of VKDB.

## 2. Case Presentation

A term 28-day-old male, born at home, was referred by the family midwife to the emergency department for ecchymosis of his left hemiscrotum. This exclusively breastfed infant, without significant family history, had been thriving at home when the parents noted acute discoloration of his right testicle at 26 days of age. In the 24 hours prior to admission, the discoloration of his right testicle migrated to the left testicle. His parents reported that he had become progressively fussy with decreased oral intake and appeared more pale. The parents denied increased work of breathing, fevers, apnea, cyanosis, emesis, or rash.

The parents stated that he had been previously healthy. Prenatal care was provided by a midwife, and prenatal labs were reported as negative. A prenatal ultrasound performed at home by the midwife at mid-gestation was reported as normal. Pregnancy was uncomplicated, as was his spontaneous vaginal delivery. The infant did not receive vitamin K prophylaxis at birth, though his mother was taking oral vitamin K, a prenatal vitamin, and herbal supplements daily. The baby was taken care of by his parents, who were Mennonite. There was no consanguinity. The baby's father had flu-like symptoms the week of his admission; otherwise there had been no known sick contacts.

**Figure 1:**
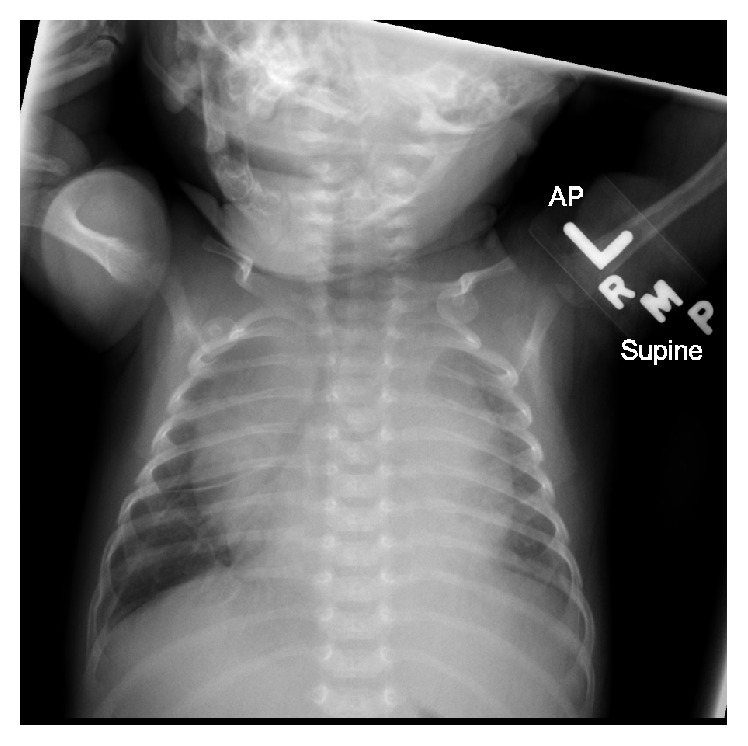
Anterior-posterior (AP) chest x-ray demonstrating marked enlargement of the cardiothymic silhouette.

On arrival to the newborn intensive care unit, the infant appeared lethargic, pale, and jaundiced and had evidence of oozing from peripheral IV attempts. His left hemiscrotum was purpuric; otherwise, there was no evidence of bruising or petechiae. He had increased work of breathing with retractions and had a normal cardiac and abdominal exam. He had no dysmorphic features and other than lethargy had an appropriate neurological exam. An anterior-posterior (AP) chest X-ray (Figure 1) performed in the emergency department demonstrated marked enlargement of the cardiothymic silhouette, which prompted further evaluation. An echocardiogram revealed a large anterior mediastinal mass with bilateral pleural effusions. A complete metabolic panel was positive for a mild indirect hyperbilirubinemia (TSB 10.1, DB 0.4; normal range TSB <11.5 mg/dL, DB 0–0.6 mg/dL) and negative for metabolic acidosis or transaminitis. A complete blood count and coagulopathy studies were significant for anemia (Hgb 8.4, Hct 24.2, normal range Hgb 10.5–14.8 g/dL, Hct 30.0–40.9%), mildly low platelets (143,000, normal range 150–500 10^3^/*μ*L), a markedly abnormal prothrombin time (greater than 80, normal range 13.2–16.5 seconds), a profoundly abnormal partial thromboplastin time (greater than 250, normal range 29–48 seconds), and a normal fibrinogen (376, normal range 150–400 mgs/dL). Based on the severely abnormal coagulation studies, the patient was treated acutely for suspected vitamin K deficiency related bleeding. The patient received three IV doses of vitamin K, packed red blood cells (60 mL/kg total), fresh frozen plasma (15 mL/kg), and platelets (15 mL/kg). Within 6 hours, the infant's hematologic indices normalized and his clinical bleeding resolved.

**Figure 2:**
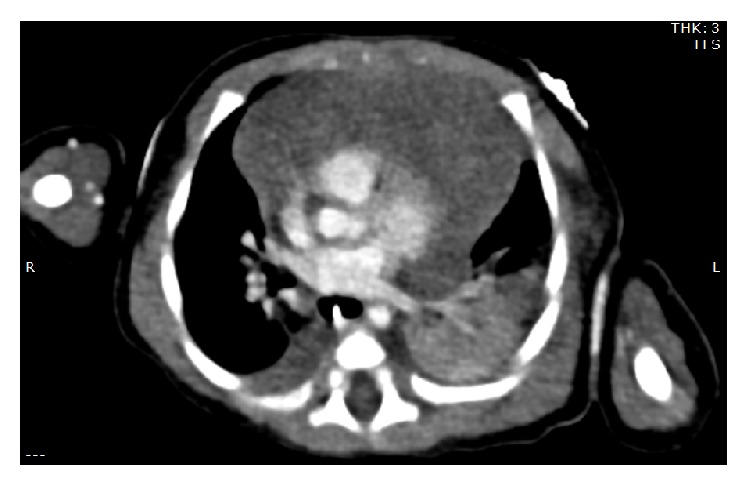
CT of chest/abdomen/pelvis demonstrated a homogeneously enhancing anterior mediastinal mass which maintained thymic contour, with mass effect upon the vessels and airway.

Despite resolution of the coagulopathy, the anterior mediastinal mass remained. The differential diagnosis included venous or lymphatic malformations, thymic enlargement, or rarer tumors. Therefore, further diagnostic imaging of the anterior mediastinal mass was pursued. A chest ultrasound revealed a solid mass without cystic components, making an infantile lymphangioma unlikely. A CT of chest/abdomen/pelvis (Figure 2) demonstrated a homogeneously enhancing anterior mediastinal mass which maintained thymic contour, with mass effect upon the vessels and airway. The homogeneity of the mass was thought to be inconsistent with the typical appearance of a germ cell tumor. While the infant's coagulopathy and borderline thrombocytopenia initially raised concern for Kasabach-Merritt phenomenon (KMP), no significant vascularity was demonstrated on the CT imaging, making the diagnosis of kaposiform hemangioendothelioma with associated KMP less likely.

**Figure 3:**
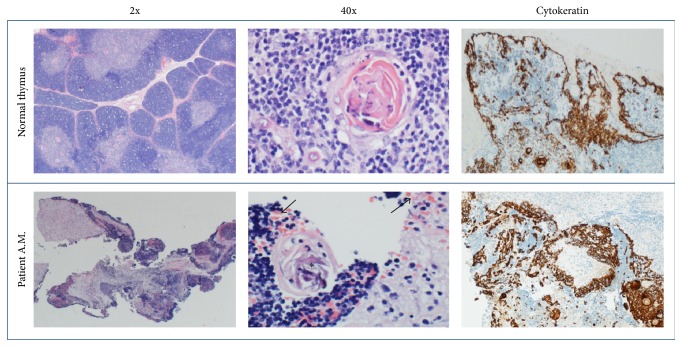
Representative histology. Comparison of core biopsy specimen from the patient's mediastinal mass with histology of normal thymus. The 2x view demonstrates overall preservation of cortical and medullary regions, with the suggestion of increased RBC's in the patient sample compared to the normal specimen. This is better seen on the 40x view (arrows) and is consistent with hemorrhage into the thymic tissue. Asterisks mark normal Hassall's corpuscles, which can be seen in normal tissue and in cases of thymic hyperplasia. Cytokeratin staining in the patient is of normal intensity and distribution, ruling out the differential diagnosis of thymoma.

The infant underwent ultrasound-guided biopsy of his mediastinal mass on the hospital day following admission once his coagulopathy had resolved. This biopsy was critical in making the diagnosis of spontaneous thymic hemorrhage. Representative histology of the mass is shown in Figure 3. The patient's tissue was consistent with normal thymic ultrastructure with Hassall's corpuscles noted and slightly increased in number compared to normal tissue. Cytokeratin staining was performed and showed no increase in intensity or aberrant distribution, ruling out the differential diagnosis of thymoma. Many red blood cells were noted in the tissue sample, and the biopsy specimen was grossly bloody as noted by the proceduralist. Flow cytometry revealed immunophenotype of immature T cells consistent with normal cortical thymocytes. In light of these pathologic findings as well as the medical history and clinical response to vitamin K and FFP, the final diagnosis for this patient was thymic hemorrhage secondary to vitamin K deficiency, with possible component of thymic hyperplasia. The patient's respiratory distress resolved over a few days and he was discharged home on hospital day #7 with follow-up by his general pediatrician.

## 3. Discussion

Newborns, in particular those who are exclusively breastfed, have multiple risk factors for VKDB. First, placental transfer of vitamin K is limited with extremely low cord concentrations (<0.05 *μ*g/L) compared to adult levels [1]. Second, infant liver reserve levels are substantially lower than adult levels [2]. Third, infant production of vitamin K is reduced secondary to immature or altered gut flora [3]. Lastly, vitamin K content in breast milk (1 *μ*g/L) is significantly lower (60 *μ*g/L) than found in formula [4].

These risk factors predispose infants to develop VKDB, classified by the age of onset. Early VKDB occurs in the first 24 hours of life but typically only is seen in infants whose mothers take medications that interfere with vitamin K metabolism (e.g., anticoagulants, anticonvulsants, or antituberculosis drugs). Classic VKDB occurs between 2 and 7 days of life and is primarily secondary to inadequate feeding. Late VKDB arises between day 8 and 6 months of life, with peak incidence between 3 and 8 weeks. This form nearly always occurs in exclusively breast-fed infants and is often associated with undiagnosed hepatobiliary dysfunction impairing absorption of vitamin K [1].

To prevent all forms of VKDB, administration of intramuscular vitamin K at birth has been a standard practice in the United States since 1961 when the American Academy of Pediatrics' recommended the injection for all newborns [5]. Without prophylaxis, early and classical VKDB have an incidence as high as 6% and 2% of live births, respectively [3, 6]. The relative risk for developing late VKDB was estimated at 81 times greater among infants who did not receive prophylaxis [7]. However, authors speculate these estimates are lower than the actual figures because the number of exclusively breastfed infants who do not receive intramuscular vitamin K are unknown. Surveillance programs in different countries, including the British Isles, Sweden, Switzerland, and Germany, demonstrate that the IM injection of 1 mg of vitamin K is more effective at preventing late VKDB than when given by the oral route [8].

The clinical presentation of early and classic VKDB is variable, with bleeding most commonly occurring from the gastrointestinal tract (53%) and umbilicus (23%) [9]. The main distinguishing feature of the late syndrome is the higher frequency of intracranial hemorrhage (ICH). An analysis of 131 published cases of late VKDB revealed 63% had presented with severe ICH, with 14% mortality and 40% long-term neurological morbidity among surviving infants [10]. In 2013, 4 cases of late VKDB were reported at a children's hospital in Tennessee, of which 3 suffered ICH [11]. Three of the infants were born at major area hospitals and one was born at home. In each case, parents had declined intramuscular vitamin K prophylaxis.

Given the potential catastrophic consequences of VKDB and the attenuation of the disease with treatment, rapid diagnosis is paramount. A confirmed case fulfills the following diagnostic criteria [8]: (1) elevation of the prothrombin time (PT) ≥ 4 times the laboratory limit of normal; (2) normal or elevated platelet count, normal fibrinogen, and absent fibrin degradation products (excludes disseminated intravascular coagulation); and (3) PT returning to normal after vitamin K administration. Once diagnosed, infants who present with a non-life-threatening bleeding should receive a single 1-2 mg intravenous dose of vitamin K_1_ (synonyms include phylloquinone and phytonadione) [8]. For life-threatening bleeding presentations, additional treatment with fresh frozen plasma (at a dose of 10–15 mL/kg) may be necessary [12].

Despite the most common manifestations of VKDB outlined above, our patient's thymic hemorrhage is an exceedingly rare clinical presentation. Our literature review identified four case reports of neonatal thymic hemorrhage [13–16]. Three of the infants did not receive Vitamin K prophylaxis at birth. Additionally, one infant with both true thymic hyperplasia and spontaneous thymic bleeding was described [17]. In all of these infants, respiratory distress in the context of imaging revealing an anterior mediastinal mass were shared features. The combination of the ultrasound and CT findings for the neonate in our case helped deter the team from performing a thoracotomy given how it narrowed the differential diagnosis.

In the present case, the overwhelming concern was the anterior mediastinal mass. Although the vitamin K deficiency was diagnosed and corrected early in the infant's hospitalization, the work-up of the anterior mediastinal mass continued. While the differential diagnosis included spontaneous hemorrhage into normal thymic tissue, malignancy or vitamin K associated bleeding into benign or malignant tissue could not be ruled out.

Anterior mediastinal masses in neonates and infants could represent venous or lymphatic malformations and thymic enlargement (whether due to thymic hyperplasia, thymic cysts, thymoma, or thymic hemorrhage). Rarer possibilities include germ cell tumors (primarily teratomas), neuroblastoma, rhabdomyosarcoma, lymphoma, Langerhans cell histiocytosis, or Wilm's tumor [18]. However, coagulopathy as an additional clinical feature narrows the spectrum of possibilities to primarily venous/lymphatic malformations and (less likely) lymphomas [19–21].

Venous and lymphatic malformations represent a diverse category of rare lesions that are nevertheless most commonly described in neonates and infants. The most common of these are infantile lymphangiomas (formerly known as cystic hygromas) and kaposiform hemangioendotheliomas (KHE). Lymphangiomas are benign congenital malformations characterized by proliferation of normal lymphatic tissue and are frequently cystic in nature [22]. They are commonly diagnosed in children less than 2 years of age and most frequently arise in the head/neck or axillae; only 1-2% are found in the mediastinum. They can be incidentally found or diagnosed due to respiratory compromise or recurrent superinfection of the abnormal cystic tissue. Kaposiform hemangioendothelioma is an aggressive vascular tumor with incidence of approximately 1 in 100,000 children, with 93% of cases diagnosed before the age of one year [20]. It can arise anywhere in the body. While intrathoracic lesions are among the rarest, this location dramatically increases (~18-fold) the risk of the major complication of KHE, which is termed Kasabach-Merritt phenomenon (KMP) [20]. KMP is thought to arise from tortuosity of the abnormal vessels in the tumor, causing trapping of platelets and secondary thrombocytopenia. Associated platelet activation then causes secondary elevation in D-dimer and hypofibrinogenemia, mimicking disseminated intravascular coagulation (DIC), often with abnormalities in PT and PTT values. Amelioration of the KMP is achieved by targeted therapy designed to cause regression of the tumor itself. Pathologically, KHE is diagnosed by its characteristic infiltrative nature, presence of spindled epithelial cells, microthrombi, and lymphatic endothelial markers such as CD34 and (variably) factor VIII-related antigen [23]. Our patient's platelet count was low normal and the fibrinogen level was in the normal range, ruling out DIC and KMP.

Primary thymic enlargement represents about 2–4% of all pediatric mediastinal masses and can occur secondary to a variety of etiologies [18]. Thymic hyperplasia is among the most common cause of enlarged thymus in younger children and is defined as increase in size and weight of the gland without change in its normal histology [24]. The true incidence is unknown, as many cases are diagnosed incidentally without associated symptoms [17]. Lymphofollicular hyperplasia is another potential cause of an abnormally large thymus and is characterized histologically by increased number and size of germinal centers. It is often associated with autoimmune disease, particularly myasthenia gravis [17, 24]. In addition to histologic examination, flow cytometry can help differentiate these two etiologies, as well as evaluating T-lymphoblastic lymphoma [24]. Thymic cysts can arise as remnants of the thymopharyngeal duct or can be acquired via degeneration of Hassall's corpuscles [17]. Finally, thymomas are indolent tumors of the thymic epithelium, most commonly seen in middle age and very rare in children [25]. Only 50 cases have been described in the pediatric literature, with patients diagnosed at a variety of ages and tumor stages [25]. Proliferation of epithelial cells, as demonstrated pathologically by cytokeratin staining, is diagnostic [25].

While lymphoma is a common cause of mediastinal mass in older pediatric patients [18], it is exceedingly rare in infants less than one year of age, with estimated incidence of 2.5 cases per million or less [26, 27]. Those case reports that exist typically describe aggressive disease with leukemic and other organ-system involvement and suggest that rare cancer predisposition syndromes such as ataxia-telangiectasia may be responsible for the young age at presentation [19, 28, 29].

## 4. Conclusion

It is crucial as a medical provider to be aware of the varied presentation of VKDB in the neonatal period. Although they are a relatively rare constellation of signs and symptoms, a nonconsumptive coagulopathy, respiratory distress, and an anterior mediastinal mass should prompt the work-up of acute thymic hemorrhage. Vitamin K deficiency bleeding should be suspected and treated as an emergency in this situation. As in our reported patient, prompt recognition and diagnosis can lead to rapid clinical resolution and avoidance of thoracotomy. Our case also led to an educational opportunity on the critical importance of vitamin K prophylaxis at birth.
